# Congenital Vallecular Cyst Causing Airway Compromise in a 2-Month-Old Girl

**DOI:** 10.1155/2015/975859

**Published:** 2015-07-09

**Authors:** Amal Faisal AlAbdulla

**Affiliations:** ENT Department, BDF Hospital, P.O. Box 28743, West Riffa, Bahrain

## Abstract

Congenital vallecular cyst is a rare entity and may present with acute airway obstruction. This is a case of congenital vallecular cyst presenting with airway compromise requiring immediate management. The epidemiology, pathogenesis, and clinical presentation of vallecular cysts are discussed as well as the diagnosis and management.

## 1. Introduction

Vallecular cysts present in neonates or infants [1]. The patient may present with symptoms such as stridor, feeding difficulty, failure to thrive, dysphonia, or respiratory distress [2]. Although these cysts are benign in nature, significant airway compromise may occur due to the increase in size. The patient may present with acute life threatening airway compromise requiring emergency airway management [1].

## 2. The Case 

**Figure 1:**
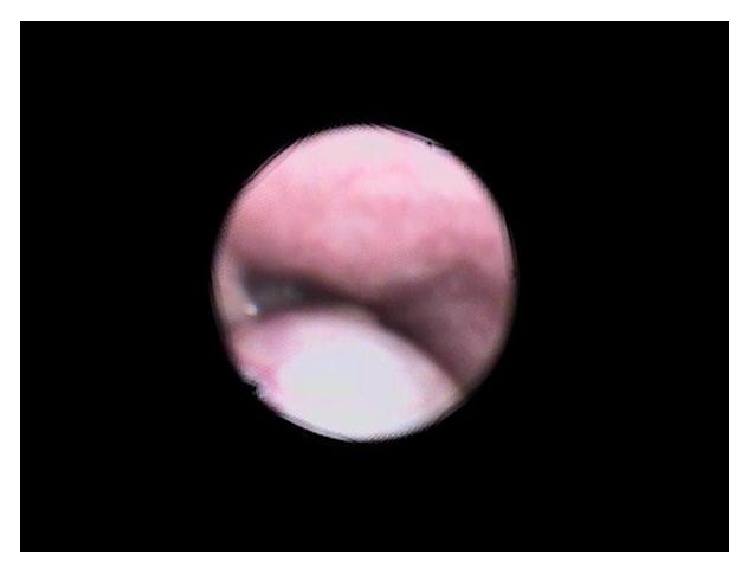
Flexible upper endoscopy showing a vallecular cyst obstructing the airway.

A 2-month-old girl was referred from the general practitioner to the Ear, Nose, and Throat Clinic with the preliminary diagnosis of laryngomalacia. She is a full term, normal delivered baby with a birth weight of 2.8 kg. She is on both breast and bottle feeds. She presented with a history of noisy breathing for 15 days which worsened during feeding. On examination, her weight was 3.5 kg, which falls below the 3rd percentile on the growth chart. She was afebrile and the rest of the vitals were normal. She was able to maintain an oxygen saturation of 96% at room air. She had inspiratory stridor with suprasternal and intercostal retraction. Flexible upper endoscopy showed a cystic lesion in the vallecula, about 1 cm in size. The cyst was pushing the epiglottis backward over the glottis and compromising the airway (Figure 1).

**Figure 2:**
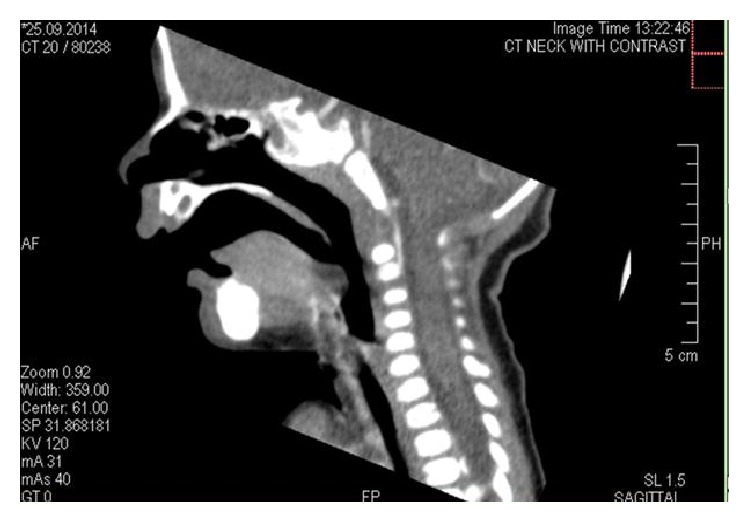
Sagittal CT scan showing a retroglossal fairly defined rounded nonenhancing hypodense cystic lesion measuring about 9 mm, seen projecting into oropharynx.

**Figure 3:**
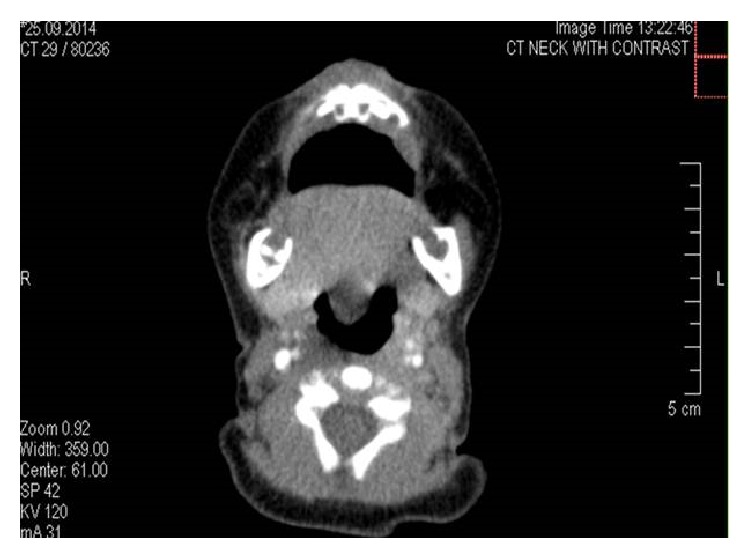
Axial CT scan showing a retroglossal fairly defined rounded nonenhancing hypodense cystic lesion measuring about 9 mm, seen projecting into oropharynx.

**Figure 4:**
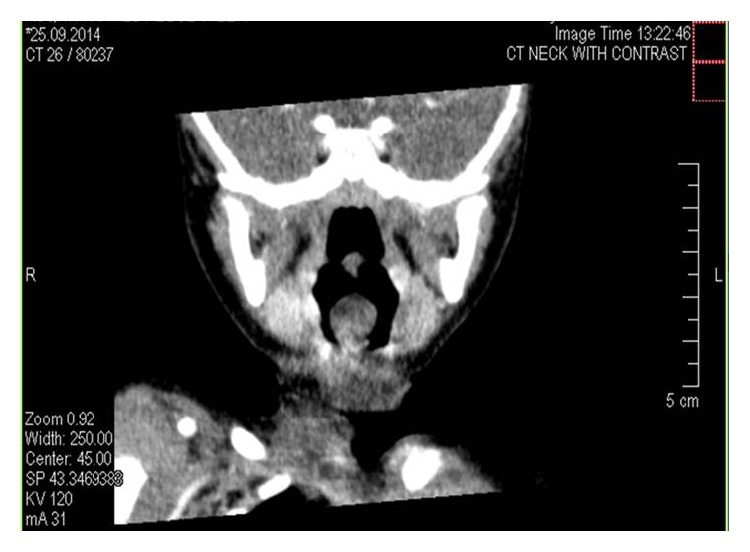
Coronal CT scan showing retroglossal fairly defined rounded nonenhancing hypodense cystic lesion measuring about 9 mm, seen projecting into oropharynx.

CT scan imaging was done on the same day and reported a retroglossal fairly defined rounded nonenhancing hypodense cystic lesion measuring about 9 mm, seen projecting into oropharynx. No soft tissue component was noted. A differential diagnosis of thyroglossal or retention cyst was suggested and clinical correlation was advised (Figures 2, 3, and 4).

Thyroid function test was done and showed normal results.

The patient was taken to the operating theater and a diagnostic direct laryngoscopy under general anesthesia demonstrated a cystic mass in the vallecula. The cyst originated from the lingual surface of the epiglottis and partially obscured the laryngeal inlet. The aryepiglottic folds, vocal cords, and subglottic region were normal, excluding other associated laryngeal pathologies. Laryngeal intubation was possible and tracheostomy was not needed. A microscope was used to magnify the surgical field and optimize visualization. The cyst was aspirated and contained a clear, transparent fluid. Aspiration was followed by partial cyst excision using microlaryngeal instruments. The cyst was grasped using a cups forceps and partially excised using micro laryngeal scissors. There was minimal bleeding from the excision margins. This was controlled by applying mild pressure using absorbent cotton gauze balls. The remaining cyst margins were then cauterized using electrocautery.

Histopathology showed a benign cyst underneath the stratified squamous epithelium and underlying edematous stroma. The cyst is lined by flattened squamous epithelium, devoid of contents. There was no inflammation or malignancy and a final diagnosis of benign supraglottic cyst was given.

Microbiology culture of the aspirated fluid grew coagulase negative* Staphylococcus aureus*. The organisms were resistant to penicillin, cloxacillin, and erythromycin and sensitive to trimethoprim sulfamethoxazole, tetracycline, and cephalexin. The patient was started on the appropriate antibiotic treatment according to the sensitivity results.

**Figure 5:**
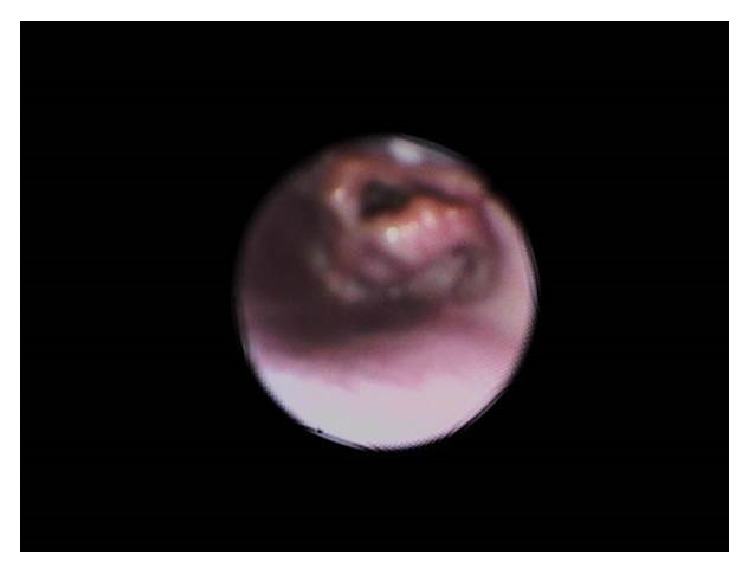
Postoperative view of the larynx using flexible upper endoscopy showing complete resolution of the cyst and patent airway.

Postoperative follow-up showed complete recovery and at 3 months of follow-up the patient had no stridor and was thriving well. A flexible upper endoscopy showed no evidence of cyst recurrence and the laryngeal inlet was clearly visualized (Figure 5).

## 3. Discussion

Vallecular cysts have been described as retention cysts which form due to ductal obstruction of the mucous glands or minor salivary glands in the vallecula and tongue base [2].

The lingual surface of the epiglottis has been described as the most common location [3]. This is supported by a 10-year review of 238 patients by DeSanto et al., where 52% of laryngeal cysts were found to arise from the epiglottis [4]. Similarly, this was found in the patient described in this report. Flexible upper endoscopy showed the cyst originating from lingual surface of the epiglottis.

Vallecular cyst is a rare entity and accounts for 10.5% to 20.1% of all laryngeal cysts [1, 5]. Tsai et al. reported an incidence of 5.3 cases/100,000 live births [6] and Prowse and Knight reported an incidence of 3.49 cases per 100,000 live births [7]. Vallecular cysts present in neonates and infants with median ages at diagnosis of 3.0 and 40.0 days [1, 4]; however, there have been reports of vallecular cysts in older children and adults [2, 5, 8, 9]. The patient described in this report presented at 2 months of age.

The usual presenting symptoms include stridor, feeding difficulty, failure to thrive, dysphonia, or respiratory distress [2, 10]. Although these cysts are benign in nature, significant airway compromise may occur due to the increase in size. The patient may present with acute life threatening airway compromise requiring emergency airway management [2]. Fatal or near fatal cases have also been reported [2].

In comparison, the patient in this report presented with stridor which worsened during feeding. She had poor weight gain and her weight fell below the 3rd percentile.

The clinical presentation of vallecular cyst may be further complicated by the presence of coexistent laryngomalacia which will influence the surgical plan of management for the patient [6]. In this reported case, there was no evidence of laryngomalacia and the symptoms resolved after surgical management of the vallecular cyst.

In one case report, there was total absence of any functioning thyroid tissue which was detected by the thyroid scan [11]. Thyroid screening and thyroid scan are advisable for all patients with vallecular cysts to rule out the lingual thyroid.

Thyroid function test was done for the patient in this report and was found to be normal.

Other differential diagnosis included cystic hygroma, lymphangioma, haemangioma, teratoma, hamartoma, dermoid cyst, thyroid remnant cyst, and thyroglossal duct cyst [11–13].

Histologically, vallecular cyst is lined with squamous epithelium and contains respiratory epithelium with mucous glands [13]. Similarly, histopathology results of the patient in this report showed a cyst lined with flattened squamous epithelium.

The initial radiologic investigation may include the neck ultrasound which will confirm the cystic nature of the lesion. However, to more accurately delineate the size and extent of the cyst, CT scan and MRI are necessary [14]. On CT scan, vallecular cyst shows low-density attenuation. On MRI, the cyst appears hypointense on T1 and hyperintense on T2 and shows no enhancement after injecting intravenous gadolinium [13]. CT scan was done for patient in this report and showed a fairly rounded nonenhancing hypodense cystic lesion. A differential diagnosis of thyroglossal or retention cyst was suggested and clinical correlation was recommended.

In cases where fetal vallecular cyst is suspected on antenatal ultrasound, a fetal MRI may be done. This will conclude the diagnosis and plan for perinatal intervention and parental counseling [13, 15].

Direct laryngoscopy remains as the gold standard for definitive diagnoses of laryngeal cysts [13]. Direct laryngoscopy was done for the patient in this report which clearly demonstrated the cystic nature of the mass, its origin, and extent.

Various surgical techniques have been described for the management of vallecular cysts; these include aspiration, marsupialization, and excision [16, 17]. Marsupialization of the cyst is the preferred treatment [18]. Different modalities have been used for this purpose; these include electro cautery, CO_2_ laser, coblation, tonsillar snare, microdebrider, or microlaryngoscopic instruments [2, 11, 18, 19]. Compared to complete excision, the recurrence rate after marsupialization has been reported as negligible [18]. Aspiration of the cyst was done for this reported case followed by partial cyst excision using microlaryngeal instruments. The remaining cyst margins were then cauterized using electrocautery. The results were satisfactory with no cyst recurrence.

## 4. Conclusion

Vallecular cyst is rare; yet it may present as an acute life threatening emergency due to sudden airway obstruction. Otolaryngologist and general practitioners as well as other health care providers must be aware of this potentially fatal pathology so that timely lifesaving measures are initiated.
